# *Acinetobacter calcoaceticus*–*baumannii* Complex Strains Induce Caspase-Dependent and Caspase-Independent Death of Human Epithelial Cells

**DOI:** 10.1007/s00284-012-0159-7

**Published:** 2012-06-09

**Authors:** Sylwia Krzymińska, Hanna Frąckowiak, Adam Kaznowski

**Affiliations:** Department of Microbiology, Faculty of Biology, Adam Mickiewicz University, ul. Umultowska 89, 61-614 Poznan, Poland

## Abstract

We investigated interactions of human isolates of *Acinetobacter calcoaceticus*–*baumannii* complex strains with epithelial cells. The results showed that bacterial contact with the cells as well as adhesion and invasion were required for induction of cytotoxicity. The infected cells revealed hallmarks of apoptosis characterized by cell shrinking, condensed chromatin, and internucleosomal fragmentation of nuclear DNA. The highest apoptotic index was observed for 4 of 10 *A.* *calcoaceticus* and 4 of 7 *A. baumannii* strains. Moreover, we observed oncotic changes: cellular swelling and blebbing, noncondensed chromatin, and the absence of DNA fragmentation. The highest oncotic index was observed in cells infected with 6 *A.* *calcoaceticus* isolates. Cell-contact cytotoxicity and cell death were not inhibited by the pan-caspase inhibitor z-VAD-fmk. Induction of oncosis was correlated with increased invasive ability of the strains. We demonstrated that the mitochondria of infected cells undergo structural and functional alterations which can lead to cell death. Infected apoptotic and oncotic cells exhibited loss of mitochondrial transmembrane potential (ΔΨ_m_). Bacterial infection caused generation of nitric oxide and reactive oxygen species. This study indicated that *Acinetobacter* spp. induced strain-dependent distinct types of epithelial cell death that may contribute to the pathogenesis of bacterial infection.

## Introduction

Bacteria of the genus *Acinetobacter* are widespread in nature. Some of them are a part of human skin flora and are important opportunistic pathogens that cause hospital-acquired infections [25, 30]. The prevalence ranges from 2 to 10 % of all Gram-negative bacterial infections in Europe [23]. The genus currently contains 33 named and unnamed genomic species. *A. baumannii*, *A. calcoaceticus*, unnamed genospecies 3 and 13 TU are genotypically closely related and phenotypically difficult to distinguish. For this reason, it has been proposed to group those species as *Acinetobacter calcoaceticus*–*baumannii* complex (Acb) [30]. They include species with the highest prevalence in clinical specimens [14, 30, 37]. They are most commonly associated with hospital-acquired infections and they account for about 75 % of *Acinetobacter* spp. isolated from clinical specimens. The isolates are usually multiresistant, requiring complicated therapy. The strains are responsible for nosocomial bacteremia, hospital and community-acquired pneumonia, endocarditis, skin, soft tissue, and urinary infections [18, 30, 33]. Many infections can be severe with mortality rates from 26 to 70 % [30].

Although the bacteria are associated with increased hospital outbreaks, little is known about their virulence factors. Outer membrane proteins of *Acinetobacter* spp. (OmpA) and lipopolysaccharide (LPS) could play an important role in its pathogenicity, resulting in lethality for mice and cytotoxicity of phagocytic and epithelial cells [6, 18]. Choi et al. [6, 7] have suggested that OmpA of *Acinetobacter baumannii* (AbOmp) is responsible for adherence, invasion of epithelial cells during the colonization. They have also suggested that the protein induces cytotoxicity and apoptosis of epithelial cells through cell surface death receptors and caspases activation. LPS is involved in a strong inflammatory response, production of cytokines and tumor necrosis factor alpha [30].

Many bacterial pathogens have developed specific strategies to suppress the effective antimicrobial response of non-phagocytic cells to avoid the host innate immune defence. The ability of bacterial pathogens to promote host cell death may be important for bacterial survival, escape from the host defence and is implicated in the mechanism of pathogenesis of a variety of infectious diseases [24, 25]. Apoptosis is a form of programmed cell death regulated by cellular signaling cascades that avoid eliciting inflammation [11]. Based on morphological criteria, cell death associated with cellular and organelles swelling and blebbing has been termed as oncosis. Necrosis is the final stage of cellular disintegration which leads to cell lysis [25, 27, 36].

The mechanism of the Acb complex strains pathogenesis has not been clearly defined. During host infection, epithelial cells are the first that the bacteria interact with. Therefore, we focused on the interaction of Acb with the cells. We chose HEp-2 cells as a well established and frequently used in vitro model for studying interactions of bacteria and human cells [12]. We determined the relationship between adhesion and invasion of epithelial cells, cytotoxic activity, production of nitric oxide (NO) and reactive oxygen species (ROS), and cell death induced during bacterial infection.

## Materials and Methods

### Bacterial Strains

Seventeen Acb strains were isolated from various specimens (wounds, aspirates, urine, catheter, sputum, abdominal cavity fluid) of hospitalized patients of intensive care units, between February 2006 and October 2010 (Table 1). They were identified with the API 20 NE system (bioMérieux). They were grown in trypticase soy broth (TSB, Difco) or agar (TSA). The strains were maintained at −75 °C in TSB containing 50 % (v/v) glycerol. All strains were gentamicin-sensitive with MIC below 50 μg/ml. A non-pathogenic *Escherichia coli* K-12 C600 strain was used as the negative control.

**Table 1:** Species of Acb complex used in the study

*Acinetobacter* species (Number of strains)	Source of origin	Strain No
*A. calcoaceticus* (10)	Secretion	MPU M5, 9, 12, 13, 17
	Urine	MPU M6, 7, 8, 19
	Medical device	MPU M4
*A. baumannii* (7)	Urine	MPU M16
	Wound	MPU M21
	Medical device	MPU M22, 24
	Secretion	MPU M20, 23, 25

MPU M—collection of *Acinetobacter* spp. strains in the Department of Microbiology Adam Mickiewicz University in Poland

### Epithelial cell line

Human laryngeal epithelial cells, HEp-2 were cultured in Eagle minimum essential medium (GM, Sigma) supplemented with 2 mM glutamine, 1000 IU/ml of penicillin G, 100 μg/ml of streptomicin, 1 mg/ml of nystatin, and 5 % fetal calf serum (FCS, Sigma) The cells were seeded with 100 μl of suspension in number 2 × 10^5^ cells per well and incubated at 37 °C in the atmosphere with 5 % CO_2_ [21].

### Infection Conditions

A monolayer of HEp-2 cells with FCS was infected with bacteria cells at the multiplicity of infection (MOI) of 100 for 90 min at 37 °C. Then the cells were washed twice with PBS and incubated with GM containing 200 μg/ml of gentamicin for 2 h to kill extracellular bacteria. Then the infected monolayer was incubated with GM without antibiotics for 24 and 48 h [7].

### *Acinetobacter* spp. Adhesion and Invasion of HEp-2 Cells

Bacterial adhesion and invasion were quantified using gentamicin protection assay [21, 22] with modifications. Epithelial cells grown in 6-well plates (Nunc) were infected with the bacteria at the MOI of 100 for 2 h. The HEp-2 monolayer where the invasion was studied was incubated with GM containing 200 μg/ml of gentamicin for 2 h at 37 °C to kill extracellular bacteria. The cells where the invasion and adhesion were studied were incubated with GM without the antibiotic for 2 h. Next, the cells were washed three times with PBS and lysed with the medium containing 0.01 M NaH_2_PO_4_, 0.1 % Tween 20 (v/v), 0.025 % trypsin (w/v) pH 8.0 for 30 min. The total number of cell-associated bacteria (intracellular plus surface-adherent) and the intracellular bacteria was determined by plating tenfold serial dilutions of the lysate onto TSA. The number of attached bacteria was determined by subtracting the number of intracellular bacteria following the invasion from the total number. The results were expressed as the adhesion index (AdI), i.e., the mean total number of colony-forming units (CFU) associated bacteria per well (2 × 10^5^ HEp-2 cells). Invasion activity was expressed as the invasion index (InI) as the percentage of bacteria after gentamicin treatment in comparison to the number of those associated. The monolayer was infected separately with an invasive strain of *Yersinia enterocolitica* O:8/1B (pYV^+^) and non-pathogenic *E. coli* K-12 C600. To inhibit bacterial internalization, HEp-2 cells were treated with cytochalasin D (1 μg/ml) (Sigma) 1 h prior to infection.

### Cytotoxicity Assay

For preparing bacterial filtrates, overnight cultures in LB were incubated in TSB in a shaking incubator with agitation at 300 rpm at 37 °C for 18 h. The supernatants were centrifuged at 3,000×*g* for 20 min and sterilized through 0.22-μm pore size membrane filters Millex-GV (Millipore). Twofold serial dilutions (from 1:2 to 1:512) of culture filtrates in phosphate buffered saline (PBS, Biomed) added to the wells of tissue culture plate containing confluent HEp-2 monolayer and incubated for 24 h at 37 °C. As negative controls, the wells received non-pathogenic *E. coli* K-12 C600 filtrate and GM. The cytotoxic titer of each isolate was calculated by determining the reciprocal of the highest dilution of the culture filtrates which produced cytopathic effect [22]. The results were observed under an inverted microscope.

### Cell-Contact Cytotoxicity

To determine whether cell contact is required for Acb cytotoxicity, we used trans-well system plates with tissue culture inserts (Nunc) with the anopore membrane with pore diameter of 0.2 μm. HEp-2 cells were cultured in the lower chamber. Following this, the bacterial cells at MOI of 100 were added in the upper chamber and incubated for 5 h. The cytotoxicity was measured by means of the mitochondrial-dependent reduction of colorless [3-(4,5-dimethylthiazol-2-yl)-2,5diphenyltetrazolium bromide] (MTT, Sigma) to a blue formazan, as described previously by Krzymińska et al. [20].

### Assessment of apoptosis

Infected HEp-2 cells were stained with acridine orange (AO) and ethidium bromide (EtBr) [31]. The monolayer was detached using 0.25 % trypsin and 0.25 % EDTA in PBS, and the suspension was stained with AO (100 μg/ml) and EtBr (100 μg/ml) solution, and examined under the fluorescence microscope (Nikon Eclipse TE-2000). The percentage of apoptotic and necrotic cells represented as apoptotic (ApI) and necrotic indexes were determined by counting a minimum of 100 cells selected at random from three preparations [21]. In positive controls, the HEp-2 cell monolayer was UV-B-irradiated (180 J/m^2^).

### Transmission Electron Microscopy (TEM)

Electron microscopy was used to evaluate morphological changes in HEp-2 cells. Infected cells after 24 and 48 h were washed three times with PBS and harvested using trypsin (500 mg/ml) and EDTA (200 mg/ml) solutions. The cells were centrifuged at 1,500×*g* for 15 min, fixed with 2.5 % glutaraldehyde in 0.1 M phosphate buffer (pH 7.2) for 1 h, washed with phosphate buffer, postfixed with 1 % OsO_4_, gradually dehydrated in series of acetone, and embedded in Epon [7]. The samples were sliced into 70-nm sections, stained with uranyl acetate and examined with transmission electron microscope (JEM 1200 EXII) TEM at accelerating voltage of 80 kV.

### DNA Fragmentation Assay

To determine DNA fragmentation, as a biochemical marker of apoptosis, the nucleic acid was extracted from infected epithelial cells, as described previously [21].

### Confocal Microscopy Imaging of Mitochondrial Transmembrane Potential

To assess the effect of Acb-infection on mitochondrial transmembrane potential (ΔΨ_m_), we used potentiometric dye tetramethylrhodamine ethyl (TMRE, Sigma). TMRE fluorescence (excitation 568 nm, emission >590 nm) was visualized on a confocal laser scanning microscope equipped with a krypton–argon laser, using a Plan Apo oil-immersion objective (Nikon). In the assay, the fluorescent dye TMRE binds to mitochondria with high ΔΨ and the dye is released from the mitochondria when ΔΨ_m_ dissipates [1, 3]. We determined the fluorescence intensity at a single cell level [4]. The results were expressed in fluorescence units (FU). The average pixel intensity for each cell and the percentage of cells that exhibited a reduced level of fluorescence were assessed.

### Caspase Inhibition Assay

To determine the possible involvement of caspases in Acb-induced cell death, the HEp-2 monolayer was pretreated with the pan-caspase inhibitor z-VAD-fmk (R&D Systems) for 1 h prior to the infection with the bacteria, and the inhibitor was present throughout the experiment. After 24 h, estimation of the apoptotic and oncotic indexes in cells after staining with AO and EtBr and estimation of ΔΨ_m_ potential revealed by TMRE fluorescence were determined.

### Measurement of NO production in infected epithelial cells

NO is rapidly converted to a stable end product, nitrite (NO_2_^−^) whose concentration in culture supernatants was quantitated using the Griess reaction [10].

To inhibit NO production, 10 nM aminoguanidine (AG, Sigma), a structural analog of l-arginine, an inhibitor of inducible NO synthase (iNOS) was used [29]. A monolayer of HEp-2 cells was also incubated for 24 h with 30 μM tocopherol (Sigma), as an antioxidant. The culture medium was removed and the cells were exposed to *Acinetobacter* spp. The inhibitor and antioxidant were included in the medium throughout the experiment and mitochondrial membrane potential of infected cells was determined.

### Statistical analysis

The data were presented as mean ± standard deviation from two independent experiments performed in duplicate. A one-way analysis of variance ANOVA with Tukey’s post hoc test at the significance level *P* < 0.05 was performed. Pairwise correlations between the apoptotic index and cytotoxicity, adhesion, ΔΨ_m_, and NO, were analyzed using the bivariate linear regression model. The Pearson′s correlation coefficient was calculated. Values at *P* < 0.05 were considered as statistically significant. The statistical analysis was performed using Statistica PL software (StatSoft Inc.).

## Results and Discussion

### *Acinetobacter* spp. Adhesion and Invasion of Epithelial Cells

Quantitative determination of adhesion and invasion of epithelial cells by Acb strains was assessed by gentamicin protection assay. All strains revealed higher adhesion ability to the cells than non-invasive *E. coli* K-12 C600 and invasive *Y. enterocolitica* O:8/1B controls (Table 2). The number of bacteria adhered to the cells was between 17.7 × 10^5^ and 28.5 × 10^5^ CFU. The non-pathogenic *E. coli* K-12 C600 showed AdI 6.1 × 10^5^ CFU, whereas that of *Y. enterocolitica* O:8/1B positive control was equal to 17.6 × 10^5^ CFU. The highest adhesion indexes, from 21.4 × 10^5^ to 28.5 × 10^5^ CFU, exhibited five (50 %) *A*. *calcoaceticus* and five (71 %) *A*. *baumannii* strains.

**Table 2:** Apoptotic (ApI), oncotic (OI) indexes of HEp-2 cells infected with Acb complex strains at 24 and 48 h with and without the pan-caspase inhibitor, extracellular and cell-contact cytotoxicity, adhesion (AdI), and invasion (InI) indexes

Strain no.	ApI (%)	OnI (%)	Extracellular cytotoxic activity	Cell-contact cytotoxicity (%)	AdI [×10^5^]	InI (%)
*A. calcoaceticus*						
MPU M12	65.1^a^/61.2	11.6^c^/17.8	32^e^	71.3^f^	25.4 ^g^	0.07 ^h^
	67.1^b^/58.7	21.3^d^/16.4				
MPU M5	61.3/57.1	8.7/6.8	32	72.4	24.3	3.1
	78.6/73.1	15.4/11.6				
MPU M13	56.3/37.1	7.9/6.8	8	65.4	27.6	0.1
	67.6/42.7	18.7/14.1				
MPU M4	56.1/61.4	11.6/12.8	1	69.8	21.6	1.2
	60.3/57.1	21.4/18.7				
MPU M9	50.7/29.2	4.8/3.6	0	54.6	21.9	11.5
	59.9/31.4	9.4/7.4				
MPU M6	43.4/47.6	50.3/45.6	0	59.7	18.8	57.8
	36.1/40.7	45.1/41.3				
MPU M19	35.4/38.1	51.4/54.3	0	39.6	20.7	51.3
	44.8/39.1	53.7/49.6				
MPU M8	18.6/23.2	49.2/45.7	0	51.4	19.6	61.2
	28.3/31.2	56.1/55.8				
MPU M17	16.1/18.3	8,5/4.2	1	31.2	17.4	0.8
	29.4/31.6	12.6/9.8				
MPU M7	14.6/7.8	51.6/54.7	0	68.5	19.7	49.3
	21.4/12.7	67.9/64.2				
*A. baumannii*						
MPU M25	58.1/53.2	11.4/7.2	0	61.3	23.6	0.08
	65.4/61.7	18.9/9.7				
MPU M20	57.4/37.8	7.8/5.4	8	64.2	21.4	0.09
	65.7/39.1	18.5/14.1				
MPU M22	57.3/31.8	11.4/8.7	1	58.6	23.7	1.8
	68.4/38.7	21.7/15.3				
MPU M21	56.1/29.7	21.3/11.4	4	57.1	28.5	2.7
	66.8/49.3	28.3/21.7				
MPU M24	38.6/13.2	5.9/5.1	32	48.3	23.8	0.6
	54.8/21.7	12.9/7.8				
MPU M23	27.3/9.8	51.1/61.2	1	61.4	15.7	52.8
	38.7/19.6	59.7/68.3				
MPU M16	32.1/29.3	48.1/51.6	0	42.1	19.1	58.1
	34.7/26.8	57.5/61.2				

^a^The percentage of apoptotic cells at 24 h without pan-caspase inhibitor/with the inhibitor

^b^The percentage of apoptotic cells at 48 h after infection without the pan-caspase inhibitor/with the inhibitor

^c^The percentage of oncotic cells at 24 h without the pan-caspase inhibitor/with the inhibitor

^d^The percentage of oncotic cells at 48 h after infection without the pan-caspase inhibitor/with the inhibitor

^e^The reciprocal of the highest dilution of supernatant yielding rounding, detachment and destruction of 50 % of a HEp-2 monolayer at 24 h

^f^The percentage of cytotoxicity was determined by MTT at 5 h

^g^The mean total number of CFU associated bacteria per well

^h^The percentage of intracellular bacteria after gentamicin treatment in comparison to initial inoculum. All values correspond to the means from two experiments in triplicate

The results could be an explanation for the prevalence of Acb strains on the epithelium which is the most frequent colonization and infection site. The interaction of the bacteria with epithelial cells is the first stage of successive bacterial invasion of the host [28]. Gohl et al. [13] have suggested that thin pili of *Acinetobacter* sp. strains mediate in biofilm formation and are involved in adhesion to biotic and abiotic surfaces.

To assess the ability of an Acb to invade epithelial cells, the monolayer was infected with the bacteria and the number of intracellular was estimated. The percentage of associated bacteria that were internalized ranged from 0.07 to 61.2 % (Table 2). The invasion index of *Y. enterocolitica* O:8/1B reached 64.1 %. Four (40 %) *A*. *calcoaceticus* and two (29 %) *A*. *baumannii* strains revealed the highest invasion ability comparable to that of the invasive control. Two (20 %) *A*. *calcoaceticus* and two (29 %) *A*. *baumannii* strains showed the lowest invasive ability, as that of non-pathogenic negative control. The invasion index for *E. coli* K-12 C600 strain that invaded HEp-2 cells was 0.07 % of total adhered cells. The treatment of epithelial cells with cytochalasin D had no effect on *Acinetobacter* spp. adhesion but resulted in reduction of the bacteria uptake in the range from 89 % (±4.1 %) to 93 % (±3.9 %) after 24 h. Cytochalasin D did not affect HEp-2 cell viability.

A prerequisite for infection is the encounter between pathogenic bacteria and the host tissue. Therefore, the ability to adhere and invade host cells is important virulence factors. Choi et al. [7] have suggested that outer membrane protein A (OmpA) produced by *A. baumannii* strains is responsible for adherence to and invasion of epithelial cells. Moreover, *A. baumannii* strains produce phospholipase D (PLD) that could be a potent virulence factor that modulates bacterial invasion of epithelial cells and enhances pathogenesis in the murine model of pneumonia [15].

### Extracellular Cytotoxic Activity

Cell-free supernatants of 10 (59 %) Acb strains were cytotoxic to epithelial cells after 24-h incubation (Table 2). The cytopathic effect caused by the activity of extracellular toxins was detected by rounding and shrinkage of the cells and destruction of the monolayer. The highest titer, amounting to 32, was observed for two (20 %) *A*. *calcoaceticus* and one (14 %) *A*. *baumannii*, the lowest one (from 8 to 1) noted for three (30 %) *A*. *calcoaceticus* and four (57 %) *A*. *baumannii* isolates. The supernatants of seven (41 %) strains and *E. coli* K-12 C600, which was the negative control, were not cytotoxic to epithelial cells.

Toxin production by Acb strains is still not clearly defined. It has been demonstrated that the most potent extracellular virulence factor produced by *A. baumannii* strains is outer membrane protein A (AbOmpA) [6, 7]. It is a porin that allows for a passing of small particles in the outer membrane and induce cytotoxicity of host cells. Jin et al. [17] identified *A. baumannii* outer membrane vesicle-mediated delivery of AbOmpA to host cells, after which the proteins induced cytotoxicity. Some *Acinetobacter* spp. strains produce a capsule composed of rhamnose, glucose, mannose, and glucuronic acid [18]. Exopolysaccharide production by pathogenic bacteria is the major virulence factor that protects the bacteria from host defences, resulting in cytotoxicity for phagocytic cells. Joly-Guillou [18] have suggested that exopolysaccharide-producing strains are more pathogenic than those non-producing ones.

### Cell-Contact Cytotoxicity

The results presented in the study provided the evidence of the cell-contact cytotoxicity of Acb strains. After 3-h incubation with Acb, the cells began to round up, and a large number of them became swollen, usually detaching from the plate surface at 5 h (results not shown). We observed that all strains were cytotoxic, which required direct contact between epithelial cell and the bacteria. The contact with five (50 %) *A*. *calcoaceticus* and one (14 %) *A*. *baumannii* induced the highest percentage of cytotoxicity (Table 2). Incubation with bacterial cells longer than 6 h caused total destruction of HEp-2 monoloayer infected with seven (41 %) strains. The lowest cytotoxicity, below 40 %, was observed for two (20 %) *A*. *calcoaceticus* strains. No cytotoxicity was noticed when *Acinetobacter* spp. cells were not allowed to contact with epithelial cells in the culture inserts, which suggested that cytotoxicity is cell-contact dependent. No cell lysis was observed at 5-h incubation with culture supernatants 14 (82 %) strains, culture medium (GM), and non-pathogenic *E. coli* K-12 C600 strain.

Secretion system proteins could be responsible for the cell-contact cytotoxicity. *A. baumannii* strains possess type II and IV secretion system proteins that transfer toxins, extracellular proteases and other enzymes from bacteria to the host cells, and contributed to the bacteria cytotoxicity and pathogenesis [8, 35].

### Apoptosis of epithelial cells infected with Acb complex strains

**Fig. 1:**
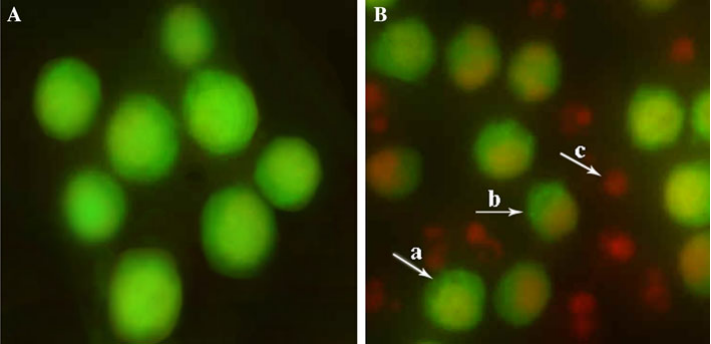
Apoptosis and necrosis of infected HEp-2 cells. The cells were stained with propidium iodide and AO (100 μg/ml) and observed in the fluorescence microscope; The cells were incubated with **A** Eagle culture medium; **B** *A*. *calcoaceticus* MPU M12; the *arrows* pointed: *a* live, *b* apoptotic, *c* necrotic cells. Magnifications: **A** ×250, **B** ×200

Live uninfected and infected cells showed green fluorescence (Fig. 1A). In contrast, red nuclei appeared in HEp-2 cells after 24 h of infection with Acb strains, indicating that EtBr and AO entered the cells (Fig. 1B). The results suggested that the infection is involved in pore formation in the cell membrane, leading to the uptake of EtBr and osmotic lysis. The highest ApI, ranging from 56.1 to 65.1 % at 24 h after infection, was observed in cells incubated with four (40 %) *A*. *calcoaceticus* and four (57 %) *A*. *baumannii* strains. The lowest ApI, between 18.6 and 32.1 %, was expressed by one (14 %) *A*. strain and three (30 %) *A*. *calcoaceticus* strains. The percentage of apoptotic cells increased at 48 h post infection (Table 2). The highest ApI ranging from 67.1 to 78.8 % was observed in HEp-2 cells infected with one (14 %) *A*. *baumanii* and three (30 %) *A*. *calcoaceticus* strains. The lowest index, ranging from 21.4 to 36.1 %, was revealed by cells infected with one (14 %) *A*. *baumannii* and four (40 %) *A*. *calcoaceticus* strains. The mean ApI of the negative control was 9.6 ± 1.8 %, whereas for the UV-irradiated positive control it reached 94.6 ± 5.1 %. The Pearson linear correlation test revealed positive correlation between the ApI and AdI of cells infected with Acb complex strains (*r* = 0.51, *P* < 0.01). The results were confirmed by treatment of HEp-2 cells with cytochalasin D, which inhibits actin polymerization and therefore, bacterial invasion of the cells. The incubation did not inhibit cell killing by apoptosis, which suggested that bacterial invasion of epithelial cells was not responsible for the cell death. Moreover, the Pearson coefficient increased for correlations between the apoptotic index and cell-contact cytotoxicity (*r* = 0.73, *P* < 0.01).

All Acb strains had low necrotic activity. Necrotic cells were characterized by their cellular swelling and orange nucleus (Fig. 1B). The necrotic indexes ranged from 4.2 ± 1.1 to 11.6 ± 4.6 % at 24 h and increased to 15.1 ± 3.8 % at 48 h.

**Fig. 2:**
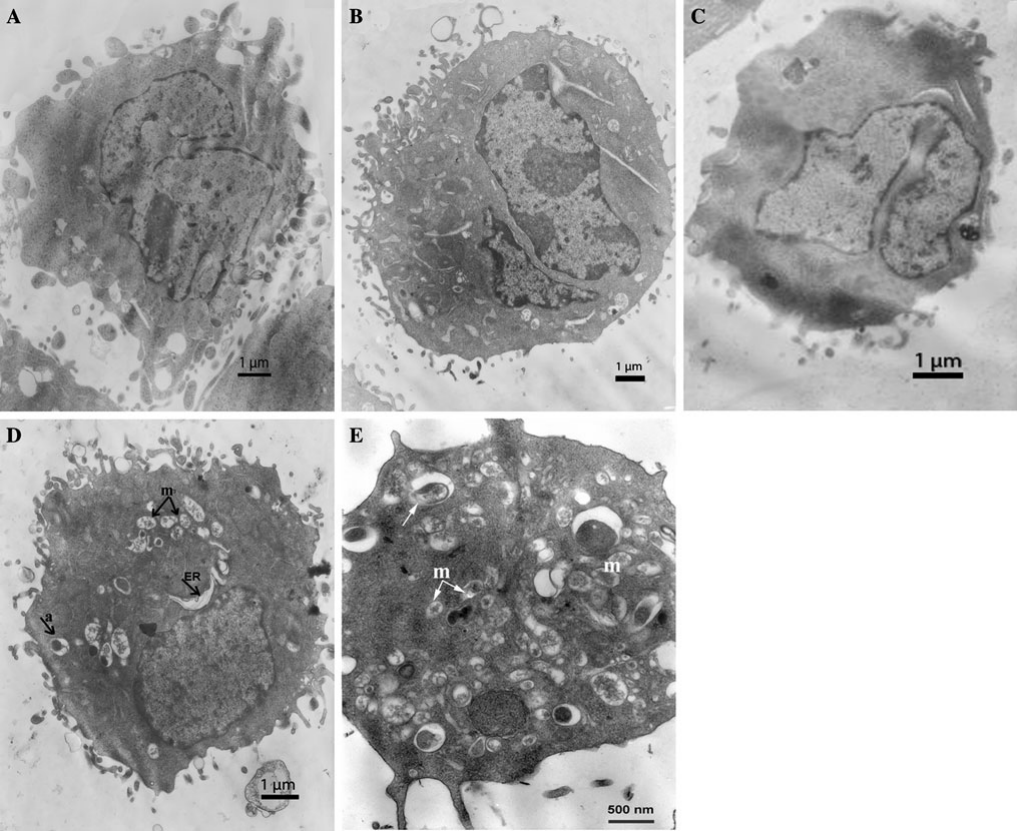
Transmission electron micrographs of HEp-2 cells infected with *Acinetobacter* spp strains. **a** Control, uninfected cell do not show remarkable morphology changes after incubation in culture medium. **b** Chromatin condensation and margination in HEp-2 cells infected with *A. calcoaceticus* MPU M12 at 24 h after infection; **c** Nuclear fragmentation; **d** Disruption of the cytoplasmic organization by *A. calcoaceticus* MPU M7 at 24 h after infection: note the dilation of endoplasmic reticulum (*ER*) elements and mitochondria (*m*), formation of autophagic vacuoles (*a*); E. Intracellular bacteria were surrounded by a membrane-bound vacuole (*arrow*), vesiculation of the cytoplasmic membrane-bound organelles, swelling and dilation of mitochondria (*m*)

To gain further insight into nuclear and cytoplasmic changes of epithelial cells caused by Acb strains, we performed electron microscopy studies compared with non-infected cells (Fig. 2a). During 24 h after infection with four *A*. *calcoaceticus* and *A*. *baumannii* strains, we observed the hallmarks of apoptosis in more than 50 % of the cells: rounding-up of the cells, retraction of pseudopodes, reduction of cellular volume, condensation with margination of chromatin (Fig. 2b), and nuclear fragmentation (Fig. 2c). The results were consistent with fluorescence microscopy observations. The analysis of intranucleosomal DNA fragmentation is used to indicate cells undergoing apoptosis. We observed patterns of low-molecular-weight DNA in cells infected with four (40 %) *A*. *calcoaceticus* and four (57 %) *A. baumannii* strains at 24 h, and with five *A*. *calcoaceticus* (50 %) and *A. baumannii* (71 %) at 48 h after infection. The pattern was observed in cells with ApI above 53 ± 3.1 %.

The results are consistent with other studies indicating that *A. baumannii* strains induce apoptosis of epithelial cells [6, 7, 34]. Smani et al. [34] have reported that an *A. baumannii* strain causes cell death of lung epithelial cells involving a perturbation of cytosolic calcium homeostasis. The strain causes release of the intracellular Ca^+2^ from the mitochondria and endoplasmic reticulum. Choi et al. [6] have observed that purified AbOmp enters the epithelial cells localized to the mitochondria and releases proapoptotic molecules cytochrome *c* and apoptosis-inducing factor (AIF).

We observed for the first that infection with Acb strains of HEp-2 cells lead to oncotic changes as cytoplasmic blebbing, with disruption of normal morphology (Fig. 2d). This included vesiculation of cytoplasmic organelles, dilation of the endoplasmic reticulum (ER) elements, and formation of autophagic vacuoles. The mitochondria from HEp-2 cells infected with the strains showed electron-dense packed cristae; in some cells, the mitochondria became swollen with completely unstructured cristae and shapes consisting of vesicle-like structures with enlarged inner space and dense inclusion bodies, providing evidence that the strains induced mitochondrial changes. We assessed the percentage of oncotic cells as oncotic index (OnI) that was determined by counting a minimum of 100 cells (Table 2). The highest OnI was observed for five (50 %) *A*. *calcoaceticus* (MPU M6, 7, 8, 17, 19) and two (28 %) *A*. *baumannii* strains (MPU M16, 23) at 24 and 48 h. Their indexes ranged from 49.2 to 51.6 % and 48.1 to 67.9 %. The strains did not cause nuclear DNA fragmentation. Interestingly, the strains had to be located intracellularly to induce oncosis, which is evident because cytochalasin D inhibited the internalization of the bacteria and prevented cell death. The oncotic indexes were reduced to the range from 9.6 to 4.2 % after treatment of the HEp-2 monolayer with cytochalasin D prior to infection. Transmission microscopic observations provided direct evidence of intracellular localization of *A*. *calcoaceticus* strains in membrane-surrounding vacuoles (Fig. 2e). Our results revealed that high invasion indexes (above 51 %), comparable to the invasive control, were consistent with the ability to induce oncosis of HEp-2 cells, showing a high level of invasion and the oncotic index. In contrast, infection with non-pathogenic *E. coli* K12C600 with low adhesion and invasion indexes did not trigger cell death.

Previous studies have suggested that host cell death may be associated with bacterial invasion. Kalischuk et al. [19] have presented that *Campylobacter jejuni* induces enterocyte oncosis, which correlates with increased invasion ability. The cell death is independent of cytolethal distending toxin expression.

### Loss of Mitochondrial Membrane Potential in HEp-2 Cells Infected with *Acinetobacter* spp. Strains

**Fig. 3:**
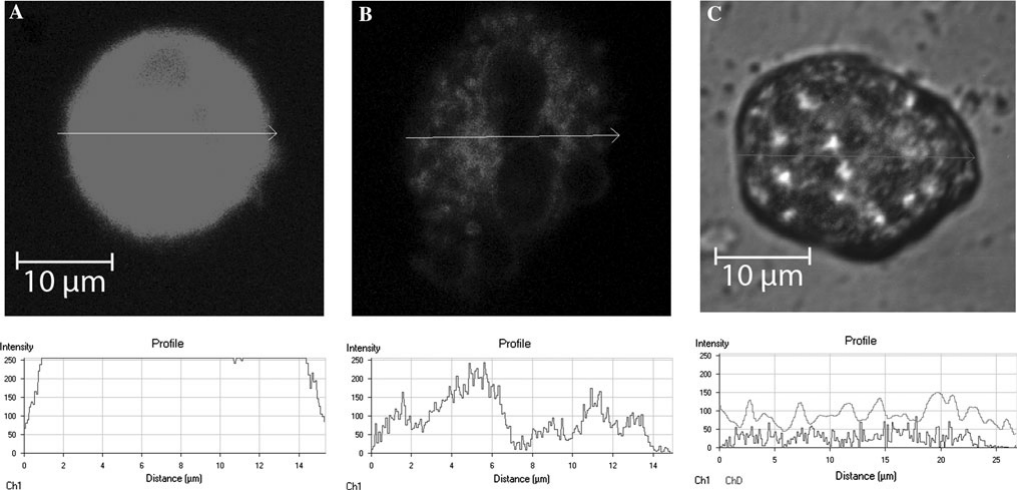
Mitochondrial membrane potential of HEp-2 cells and TMRE fluorescence intensity at a single cell level after 24-h incubation with: **a** Eagle minimum essential medium, **b** *A*. *calcoaceticus* MPU M12, **c** *A*. *calcoaceticus* MPU M7. ΔΨ_m_ was assessed after TMRE staining. The fluorescence was visualized by laser confocal microscopy

While TEM observations manifested that the mitochondria found in epithelial cells are morphologically disrupted during Acb-infection, the functional nature of these organelles within the infected epithelium remains uncertain. Therefore, we determined the effect of the strains on mitochondrial transmembrane potential (ΔΨ_m_). We observed that infected HEp-2 cells, in the range from 56.3 ± 3.1 to 89.6 ± 4.1 % exhibited a reduced level of TMRE fluorescence, compared to only 6.8 ± 2.1 % of the control ones incubated with a non-pathogenic *E. coli* strain. The number of the cells with reduced level of fluorescence increased to the range from 67.1 ± 2.8 to 97.3 ± 2.1 % at 48 h after infection. We determined the fluorescence intensity at a single cell level (Fig. 3). The average pixel intensity for each cell was assessed. We determined the membrane potential in viable, apoptotic, and oncotic cells. The fluorescence was the highest in control viable cells (Fig. 3a) in comparison with apoptotic (Fig. 3b) and oncotic ones (Fig. 3c). The control cells had mean fluorescence above 250 FU. *Acinetobacter* spp.-infected apoptotic cells revealed decline in TMRE fluorescence in comparison with the controls to the minimum level of 65 ± 27 FU at 24 h. Interestingly, the lowest values of fluorescence intensity in the range from 51 ± 22 to 24 ± 11 FU were noticed in oncotic cells. The reduction of intensity of ΔΨ_m_-sensitive dye TMRE in *Acinetobacter* spp.-infected cells indicated mitochondrial depolarization. The Pearson test revealed positive correlation between the ApI and loss of ΔΨ_m_ (*r* = −0.58, *P* < 0.01).

Mitochondria have been identified as the target of an increasing number of bacterial proteins, which are transferred to the cell during infection and play a crucial role in bacterial pathogenesis and modulation of host cell death [2, 32]. Therefore, many microbial strategies are aiming at targeting these organelles to stimulate cell death pathways. Rudel et al. [32] have suggested that loss of mitochondrial functions is a feature of both apoptotic and oncotic cell death. The disruption of ΔΨ_m_ has been established to be an indicator of mitochondrial damage. A reduction in the potential is believed to be mediated by the opening of the mitochondrial pores. Under normal circumstances, most of the pores are closed, and their opening has consequences for mitochondrial physiology, including efflux of small molecules and proteins from the mitochondrium. Strains of *A. baumannii* induced a release of proapoptotic molecules such as cytochrome *c* and AIF [6].

### Inhibition of Caspases

In cells infected with 5 (29 %) strains, the treatment with the pan-caspase inhibitor resulted in the reduction of apoptotic cells by 34.2–65.8 % after 24 h (*P* < 0.01). The results indicated that apoptosis of epithelial cells induced by the strains could be associated with the activation of caspases. In contrast, the inhibitor did not significantly affect ApI in cells infected with 7 (64 %) strains and did not prevent Acb-induced oncosis (Table 2). The results suggested that the cell death induced by the strains is independent of known caspases.

Activation of caspases could be recognized as a key element in the apoptotic process. Smani et al. [34] have reported that lung epithelial cell apoptosis induced by *A. baumannii* strain involves calpain and caspase-3 activation. However, new evidence is drawing attention to the emergent role of cell death pathways, where caspases are not involved [26]. There are some other proteases (cathepsins, calpains, granzymes, and serine proteases) that induce apoptosis in the absence of caspase activity [5]. Moreover, apoptosis could be activated by mitochondrial changes that do not require caspases. One of distinct pathways from the mitochondria is the release of the AIF from the intermembrane space. AIF induces caspase-independent formation of large (50 kb) chromatin fragments. Choi et al. [6] have suggested that outer membrane protein 38 (omp38) affects the mitochondria and releases proapoptotic molecules such as cytochrome *c* and AIF. Another mitochondrial protein that contributes to caspase-independent cell death is endonuclease G that induces DNA fragmentation in nuclei [5].

### *Acinetobacter* spp.-induced NO production in epithelial cells

We tried to define the mechanisms leading to loss of mitochondrial integrity. We observed that the infection induced a significant increase in the level of intracellular NO in epithelial cells. All strains produced increased levels of NO, significantly higher than that of the non-pathogenic control (1.1 ± 0.6 μM at 24 h, 1.5 ± 0.3 μM at 48 h). The highest NO production, 17.3–25.8 μM, was detected in cell supernatants infected with four (40 %) *A*. *calcoaceticus* (MPU M19, 8, 7, 6) strains and two (28 %) *A. baumannii* (MPU M16, 23) at 24, the concentrations decreased to 11.7–19.2 μM at 48 h for the strains. Moreover, we observed positive correlation between the apoptotic index and NO production (*r* = 0.73, *P* < 0.01).

The incubation of infected cells with aminoguanidine reduced the number of the dead cells with low TMRE fluorescence to the range from 26 to 41 %, suggesting that NO induced depolarization of the mitochondrial membrane. Treatment with tocopherol significantly inhibited Acb-induced ROS production and increased ΔΨ_m_ levels nearly as the control cells. The mean percentage of the cells with a reduced level of TMRE fluorescence decreased to 12.6 ± 4.1 (*P* < 0.01), suggesting that the loss of mitochondrial membrane potential was a consequence of oxidative stress.

Host cells death can be induced by endogenous second messengers, which are elicited in response to stress. NO is one of the factors that can trigger apoptosis [9]. However, NO involvement in the cell death induced by Acb has not yet been characterized. We observed that most (83 %) of the strains that induced the highest level of NO were isolated from urine. It has been observed that the increased concentration of free radicals in infected kidneys plays a role in the injury of renal cortical cells in uropathogenic *E. coli* infections [16]. NO can stimulate mitochondrial production of ROS, like superoxide, hydrogen peroxide, peroxynitrite, which all have proapoptotic potential. NO alone could also inhibits mitochondrial respiration, and reacts with ROS to form the particularly cytotoxic species peroxinitrite [11]. Smani et al. [34] have reported that infection of lung epithelial cells with *A. baumannii* strain induces ROS generation that leads to the cell death.

## Conclusions

Induction of host cell death is thought to play an important role in bacterial pathogenesis [25]. In the study, we provided evidence that Acb complex strains induce apoptosis of epithelial cells from an extracellular location, through adhesion to the cells and cell contact-mediated cytotoxicity. Moreover, for some strains, bacterial cell contact of epithelial cells and invasion was required for Acb-induced cytotoxicity and cell death by oncosis. The results suggested that activation of different cell death pathways in epithelial cells could constitute an important pathogenic mechanism by which the strains evade host immune defence and cause disease. The results provide new insights into the pathogenesis of Acb complex infections.
